# Establishing a genomic radiation-age association for space exploration supplements lung disease differentiation

**DOI:** 10.3389/fpubh.2023.1161124

**Published:** 2023-05-11

**Authors:** Nathan A. Ruprecht, Sonalika Singhal, Kalli Schaefer, Jappreet S. Gill, Benu Bansal, Donald Sens, Sandeep K. Singhal

**Affiliations:** ^1^Department of Biomedical Engineering, University of North Dakota, Grand Forks, ND, United States; ^2^Department of Pathology, University of North Dakota, Grand Forks, ND, United States

**Keywords:** bioinformatics, biological aging, genomics, lung disease, ionizing radiation

## Abstract

**Purpose:**

One possible way to quantify each individual's response or damage from ionizing radiation is to estimate their accelerated biological age following exposure. Since there is currently no definitive way to know if biological age estimations are accurate, we aim to establish a rad-age association using genomics as its foundation.

**Methods:**

Two datasets were combined and used to empirically find the age cutoff between young and old patients. With age as both a categorical and continuous variable, two other datasets that included radiation exposure are used to test the interaction between radiation and age. The gene lists are oriented in preranked lists for both pathway and diseases analysis. Finally, these genes are used to evaluate another dataset on the clinical relevance in differentiating lung disease given ethnicity and sex using both pairwise *t*-tests and linear models.

**Results:**

Using 12 well-known genes associated with aging, a threshold of 29-years-old was found to be the difference between young and old patients. The two interaction tests yielded 234 unique genes such that pathway analysis flagged IL-1 signaling and PRPP biosynthesis as significant with high cell proliferation diseases and carcinomas being a common trend. *LAPTM4B* was the only gene with significant interaction among lung disease, ethnicity, and sex, with fold change greater than two.

**Conclusion:**

The results corroborate an initial association between radiation and age, given inflammation and metabolic pathways and multiple genes emphasizing mitochondrial function, oxidation, and histone modification. Being able to tie rad-age genes to lung disease supplements future work for risk assessment following radiation exposure.

## 1. Introduction

Biological aging is an ever-evolving field of study. While the need exists in detaching from concepts of simple chronological age (CA), a standard cannot exist without a consensus on what is being estimated. The Klemera–Doubal (KD) method in computing biological age (BA) emerged as an early standard in 2006 as an improvement over MLR and PCA by evaluating biomarkers according to their impact on life expectancy and treating CA as another independent variable instead of as the criteria (1). Since then, great strides have been made on expanding its accuracy and building on the understanding of aging mechanisms by employing deep learning techniques for estimation (2), exploring multiomic approaches (3), and integrating for both wellness and disease predictability (4, 5). Arguably the most significant advancement in aging theory has been the development of the epigenetic clock using 353 methylation-based biomarkers as a means to estimate biological age (6). During literature review of BA estimation, a plethora of studies claim great yet unrelated success and lack of consensus for the sake of publication culture in science and academia. Before proposing yet another biological age estimator, this study instead takes a step back to a precursor requirement in identifying a reference variable to gauge relative accuracy.

Exposure to ionizing radiation (IR) is known to have similarities with aging. IR was thought to cause accelerated aging in an organism since it induced a large amount of diseases closely related to natural aging such as cardiovascular diseases, cancers, autoimmune diseases, cognitive impairment, cataracts, and a shortened life span (7). At first, the association was dismissed since radiation exposure mostly caused genetic damage and affected proliferating cells. But recent advances in understanding the overlap between the fields of aging and the biological effects of ionizing radiation (IR) are largely related to oxidative stress and inflammation, genomic instability, stem cell exhaustion, and cell senescence. In one such study, Campisi and d'Adda di Fagagna discuss the role of cellular senescence in aging and disease, including its relationship to oxidative stress and inflammation. The authors also highlight the effects of ionizing radiation (IR) on cellular senescence and the potential for IR to induce cellular stress responses, such as genomic instability and stem cell exhaustion (8). This study concludes that while there is still much to be learned about the connection among aging, cellular senescence, and IR, it is clear that these processes are closely related and require further investigation. However, limited data do not support a direct correlation between IR and changes in telomere length as reported by Sabatino et al., where IR may contribute but other factors are likely to play a more significant role (9). Along with the nine hallmarks of aging, the three processes of aging can be seen as the accumulation wear and tear, antagonistic pleiotropy, and disposable soma—we found Richardson's article to be thoroughly informative on these concepts and reviewing mentioned overlap between age and IR (10).

Biological effects of radiation are still a large field of study in dealing with dose, dose-rate, type of radiation, age of individuals, types of cells or tissues in question, underlying conditions, nutrition or lifestyle, and even calibrating measurements for these mentioned variables. While there is still some need for understanding the workforce dealing with nuclear materials, renewed interest has been seen in applications in space. Beyond low-Earth orbit (LEO), astronauts will face significantly more radioactivity than on Earth due to solar particle events (SPEs), galactic cosmic rays (GCR), and solar wind. GCR dose-rates can be 50–100 mGy/year at solar maximum and 150–300 mGy/year at solar minimum, while SPEs can fluctuate wildly and achieve 1,400–2,837 mGy/h. On top of high levels of radiation, no studies have successfully emulated the intravehicular radiation spectrum that astronauts are exposed to during space travel (11, 12). While acute exposure is understood and avoided by any means, there is a gap in consensus with low-dose, chronic exposure. In 2021, the Nuclear Regulatory Commission (NRC) upheld its linear, no-threshold model as a sound basis to protect against radiation and is endorsed by the U.S. Environmental Protection Agency. The United Nations Scientific Committee on the Effects of Atomic Radiation no longer supports the model for very low radiation doses as well as disputes have been submitted to the NRC to officially move beyond such a model (13–15). However, there may exist some threshold to exceed, or evidence of radiation hormesis in the longterm, to contradict thoughts of linear, no-threshold assumptions (16–18). While there are some experimental and plant studies showing benefit from radiation exposure, it is a complex network of variables in an undeveloped field that would need a number of dedicated studies and controls that are out of scope at this time (19, 20).

Instead, we focused on gathering publicly available data from original studies that exposed blood to gamma radiation. Our hypothesis is that genomics can serve as a foundation in creating an association between effects of ionizing radiation and natural aging processes. To test this, we must make a few compromising assumptions given that previously collected data for secondary analysis will have static and limiting clinical information. Since biological age is understood to relatively follow chronological age with some variation, CA is used to assess commonality with radiation exposure (21–23). This allows us to initially study the rad-age association with genetics focus without having to implement a biological age estimator. This lays a large assumption that BA is dependent on CA which is an accepted risk for this study. Should the two rad-age variables correlate and therefore supplement effects, biomarkers can be used to indicate conditions closely related to the two separately. Cells with high proliferation rate are particularly susceptible to damaged DNA that may accumulate over time (age) or environmental factors (radiation), thus leading to using disease diagnosis as a case point (10, 24). Lungs were chosen as an initial organ of interest because of its overlap in disease with increased age or radiation damage. While findings have potential benefits to other organs or systems, this serves as a starting point for future studies.

## 2. Materials and methods

A known limitation of secondary analysis is data availability for target factors. Because of this, we use various datasets to evaluate different pieces of our intended hypothesis while maintaining control on a few variables such as organism (human), tissue (blood), radiation type (gamma rays), and dose (within CDC categories). We use two datasets of otherwise healthy individuals to study age, one dataset of radiation only, two other datasets that capture both age and radiation exposure, and finally one dataset involved with a number of lung diseases. The intent of the first five datasets is to generate a list of statistically significant genes that can be used for (a) functional pathway and disease analysis and (b) used for predictability of the various lung diseases of the sixth dataset. The rest of this section is structured to introduce the reader to these datasets, the statistical processes for evaluating age, the interaction tests for radiation and age, and applicability to predicting lung disease.

### 2.1. Data characteristics

**Figure 1:**
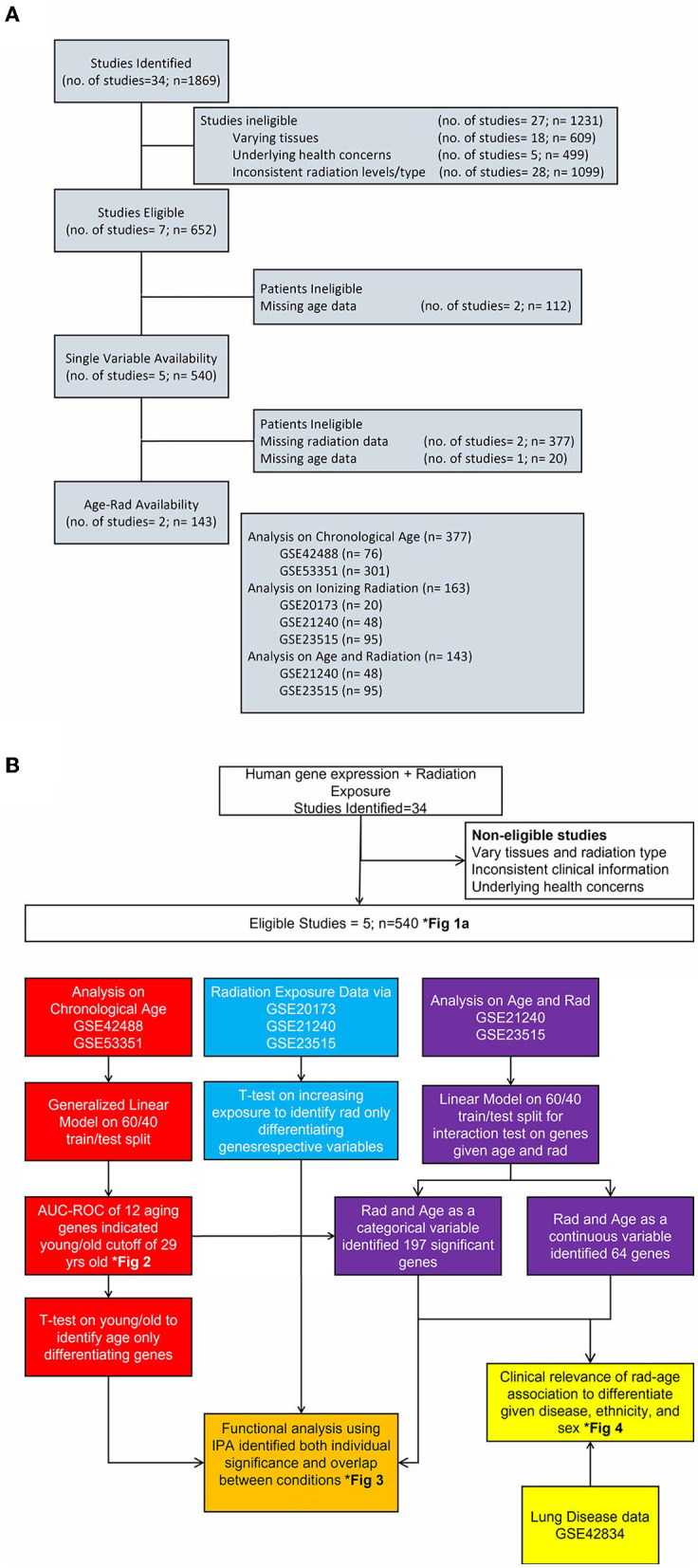
Flow of study identification and elimination. **(A)** Continued breakdown to show study consideration and assignment of original studies to categories of analysis. We show stages of dwindling down the 34 identified studies with reasoning for eliminating and eventual choosing five studies for each area of interest. **(B)** Detailed look at the completed process for this article with color-coded separation for each analysis category. This is an integrated look at our overall approach given various datasets, methodology applied, and a few results in identified figures.

Datasets from multiple repositories were evaluated for relevance and secondary analysis feasibility. While 34 sets were initially identified with 1,869 samples, eliminating factors included different tissues samples, underlying health concerns that would have unknown effects on results, inconsistent radiation levels or different types of radiation exposure, age or radiation levels not collected or part of the experiment, and trimming outliers from our final set to better capture the variance of expression. The flow of datasets evaluation as well as this article's research flow is shown in Figure 1 with clinical information on chosen data in Tables 1, 2. To best capture and combine different data, five identifiers from GEO platform GPL6480 were used that included samples exposed to a range of gamma radiation and healthy controls: GSE20173, GSE21240, GSE23515, GSE42488, and GSE53351. All datasets are studies previously conducted on human cohorts and blood samples collected for respective analysis. They are publicly available and are used here as secondary analysis for the purpose of studying the genetic interaction between age and radiation exposure. The distribution of gene expression along with the principal component analysis is calculated and provided in Supplementary material for descriptive purposes to support unbiased processing and data usage. However, for the most precise analysis, GSE identifiers were combined and processed depending on the category being evaluated using R package limma v3.52.1. Datasets were for both independent and dependent analyses of chronological age and ionizing radiation to capture a genomics-based foundation for association and indirect benefit to disease differentiation. To better reference these datasets, nomenclature follows “A” for datasets used to analyze solely age, “B” for the radiation-age interaction (or “Both”), “D” for the disease dataset, and “R” for radiation only analysis.

**Table 1:** Demographics of the five datasets we use to analyze the association between radiation exposure and aging processes.

					**Radiation**				
		**Age**				**Interaction models**			
		**GSE42488**	**GSE53351**		**GSE20173**	**GSE21240**	**GSE23515**		
		**Data-A1**	**Data-A2**	***P***	**Data-R1**	**Data-B1**	**Data-B2**	***P***	**Total**
Sex	Male	38	102	0.670	-	40	48	0.018	228
	Female	36	198		-	8	47		289
Age as a categorical variable (yrs)	Young (≤29)	16	0	0.001	-	24	24	0.823	64
	Old (>29)	58	300		-	24	71		453
Radiation exposure levels (Gy)	Control (*D*_*T*_ = 0)	74	300	-	10	24	24	0.222	432
	Low Rad (0 <*D*_*T*_ <0.5)	0	0	-	0	0	24	0.293	24
	Med Rad (0.5 ≤ *D*_*T*_ <1)	0	0	-	0	24	24	0.873	48
	High Rad (1 ≤ *D*_*T*_)	0	0	-	10	0	23	0.042	33
Gravity	0.1 g	-	-	-	10	-	-	0.076	10
	1 g	-	-	-	10	-	-		10
Smoking behavior	Smoker	-	-	-	-	-	47	0.479	47
	Non- smoker	-	-	-	-	-	48		48
	Total	74	300		20	48	95		537

**Table 2:** Demographics of the “disease” dataset to evaluate potential applications of our findings from rad-age association.

		**GSE42834**		
		**Data-D1**		
		**Train**	**Test**	**Val**	**Total**
Disease	Control	46	52	23	121
	Pneumonia	-	6	-	6
	TB	16	11	8	35
	Active sarcoidosis	15	16	6	37
	Non-active sarcoidosis	8	9	4	21
	Lung cancer	8	8	-	16
Ethnicity	Central Asia	-	-	1	1
	SE Asia	-	3	-	3
	Indian subcontinent	15	15	6	36
	Middle Eastern	-	2	-	2
	Caucasian	56	60	19	135
	African	21	22	15	58
Sex	Male	46	47	13	106
	Female	47	55	28	130
	Total	93	102	41	236

From the combined sets of GSE42488 (Data-A1) (25) and GSE53351 (Data-A2) (26), the original studies includes 377 samples taken from otherwise healthy, control patients of chronological age ranging from 21 to 69 years of age. The study that yielded the Data-A1 dataset involved collecting whole blood from 76 individuals in Japan to study peripheral blood mRNA expression. Data-A2 dataset was collected from 301 apparently healthy individuals residing in Japan to study the transcriptional profile in peripheral blood cells. Outliers were detected and excluded from Data-A1 (2 samples) and Data-A2 (1 sample). We preprocessed, combined, and removed the batch effect of studies using R package limma v3.52.1 on this data to continue with our secondary analysis of original data to empirically evaluate an age threshold between old and young patients. A recognized limitation of this approach is the region of the original studies as ethnicity is not taken into account due to data availability.

Interaction analysis was conducted on the combined sets of GSE21240 (Data-B1) and GSE23515 (Data-B2) (27), where a total of 143 originally collected samples were taken with a chronological age range from 21 to 64 years exposed to various levels of ionizing radiation to include 0 Gy (controls), 0.1 Gy, 0.5 Gy, and 2 Gy. Data-B1 was a study of peripheral blood mononuclear cells (PBMCs) from six individuals, following two different blood preservation methods, performing RNA extraction immediately or 3 h after an *ex vivo* exposure to 0.5 Gy of Cesium-137 gamma rays for 1 min totaling 48 samples. Data-B2 studied peripheral blood cells from 24 different donors (95 samples due to one sample being lost) exposed *ex vivo* to 0 Gy (controls), 0.1, 0.5, and 2 Gy at a dose rate of 0.82 Gy/min from Cesium-137 gamma radiation to study radioactive responses between sex and smoking behavior.

Combined with Data-B1 and Data-B2, GSE20173 (Data-R1) (28) was included for ionizing radiation only analysis to capture 20 additional samples collected during its original study. Data-R1 analyzed the miRNA expression profile of peripheral blood lymphocytes incubated for 4 and 24 h in normal gravity (1 g) and in modeled microgravity after irradiation with 0.2 and 2 Gy of gamma rays (five participants across four conditions). From the five datasets, Data-R1 was excluded when evaluating sex due to this information not being collected at the time of the original study being conducted. Data-B1, Data-B2, Data-A1, and Data-A2 result in 230 male and 290 female samples of varying age and radiation exposure.

Finally, we use a dataset of patients with various diseases of the lung. GSE42834 (Data-D1) (29) samples whole blood to compare patients with tuberculosis (TB), sarcoidosis, pneumonia, and lung cancer and is pre-categorized to train, test, and validation sets. While initially 356 samples were involved in the original study, missing demographics and focusing on separated data dwindles down the used data in this article to 236 samples as shown in Table 2. These data were used to explore statistical relevance of identified rad-age genes in differentiating patients with regard to their illness, ethnicity, and sex.

### 2.2. Statistical and functional analysis

To evaluate age as a categorical variable with regard to old vs. young patients, a cutoff threshold needed to be defined. While 16 known genetic indicators of age were initially of interest to analyze thresholds, four were not in the aging datasets but were included here for a thorough review: *GATA6* (30), *RIPK1* (31), *CDKN1A* (*P21*) (32), and *RIPK3* (33). The remaining 12 were available and used to analyze threshold from 21 to 65 years of age, which are as follows: *CDKN2A* (*P16*) (34), *FOXO1* (35), *SIRT1* (36), *IL6* (37), *TFAM* (38), *mTOR* (39), *TSC1* (40), *TP53* (41), *SIRT6* (42), *MLKL* (43), *ALOX15B* (44), and *TNFAIP3* (*TNF*α) (45).

As mentioned above, the raw data of Data-A1 and Data-A2 from GPL6480 were combined and processed using R package limma to produce 374 of otherwise healthy, control patients to explore age threshold cutoffs. The intent was to implement a for-loop through the range of chronological ages to evaluate each as a cutoff for age classification. During the first loop, set all samples of 21 years of age or less to the “young” category or labeled “1” and all other samples greater than those just identified to “old” or “0” class, then iterating through to 65 years of age. With these two classes, perform a student *t*-test and generalized linear model to evaluate the significant differences and predictability using those aging genes just described. A student *t*-test was conducted for each gene of interest at each age year to capture the respective *p*-value and fold change. Additionally, a generalized linear model was fit using the genes individually on each year to calculate the area under the curve (AUC) of the receiver operating characteristic (ROC) to evaluate the accuracy of the model with respect to specificity and sensitivity. For modeling, the data were split into train/test subsets using a 60/40 ratio while maintaining distribution of age as a continuous variable. Interpreting the significance and AUC-ROC of the genes led to a cutoff age used for defining young vs. old in all future analysis. As the age cutoff progressed, the number of young and old samples would change. Supplementary Figure 2A depicts our approach to accounting for an uneven distribution. With a lower cutoff, there would be less “young” samples compared to those classified as “old.” We would randomly sample patients from the larger class in order to have a 50/50 split in an effort to create an unbiased dataset from modeling. We did 1,000 permutations of this approach to get an average AUC for each gene at each age threshold. At some point, the age cutoff would reach a point where there were less “old” patients than “young,” in which case, our approach remains the same in sampling the larger patient set (young) to match the lesser (old) for a 50/50 split and run 1,000 times. This ensures an even ratio of each class while randomly sampling to capture the entire population.

For radiation classification, Data-R1, Data-B1, and Data-B2 were similarly combined and processed using the same R package and processed to produce 163 samples with controls or levels of 0 Gy exposure labeled as “control” or “0”, low/1 for 0< *D*_*T*_ <0.5 Gy, medium/2 for 0.5 ≤ *D*_*T*_ <1 Gy, and high/3 for 1 Gy≤ *D*_*T*_ exposure levels. These radiation bins fall in line with the radiation hazard scale set forth by the Centers for Disease Control and Prevention (CDC). Our low category is equivalent to a CDC Category 3, medium to a Category 4, and high to a Category 5. Since our bins were chosen based on the available data ranges for the sake of initial studies, a larger distribution will be needed to capture very low exposure that may exist. For evaluating sex, samples were the combined datasets of Data-B1, Data-B2, Data-A1, and Data-A2 with males as class 1 and females as class 0. Only datasets that included a factor of interest were used to analyze that factor, for example, Data-A1 and Data-A2 were used to study only age and had no influence on our methodology or results for studying radiation exposure. Student *t*-test was performed on the six combinations of radiation exposure and sex to capture significant genes with regard to t-statistic, FDR adjusted *p*-value, and log2FC. The results were used for pre-ranked enrichment analysis using Ingenuity Pathway Analysis (IPA). Results of the gene level analysis via *t*-test and pathway analysis via IPA are recorded and discussed.

A linearized model was implemented to conduct an interaction test between age and radiation exposure (general form of *genes*~age*rad and referred to as interaction models). As mentioned, Data-B1 and Data-B2 were processed together since the original studies collected both age and radiation information. With the age cutoff in mind, models were created for both age as a categorical (young/old) and as a continuous variable. Similarly, data were split into train/test subsets using a 60/40 ratio maintaining distribution of chronological age as a continuous variable (and therefore classes) and ionizing radiation exposure levels. The significantly changing genes with an Age*Rad *p* < 0.05 were used for functional analysis via IPA while also looking at other clinical factors.

### 2.3. Clinical applicability

The intent was to see if the genes just identified to associate aging process to radiation exposure have application potential when looking at disease along with demographic separation of ethnicity and sex. A successful finding would mean that direct association of rad-age also has an indirect association to lung diseases. Therefore, preventing/monitoring radiation exposure or aging would have the secondary benefit to preventing such diseases.

After the linear models to test the interaction between radiation exposure and age (both as a continuous and categorical variable), the combined significant gene list comprised of 234 genes. We conducted a pairwise comparison of these genes from Data-D1 to look at differentiation among ethnicity (i.e., Caucasian vs. others, African vs. others, and Indian vs. others), disease (i.e., Control vs. others, lung cancer vs. others, etc.), and sex. Again, being secondary analysis, we are limited to evaluate factors that were originally collected only. We filtered results by genes that were significantly different (*p* < 0.05) in at least two of these test cases across all train, test, and validation sets, as well as a log2FC greater than 2. With these genes that are now validated on independent analysis of demographics, we then do an interaction test to capture dependent significance using a linear model in R with the general form of *gene*~disease*ethnicity*sex. Three genes had significant differentiation and were presented for discussion.

## 3. Results

### 3.1. Chronological age threshold

**Figure 2:**
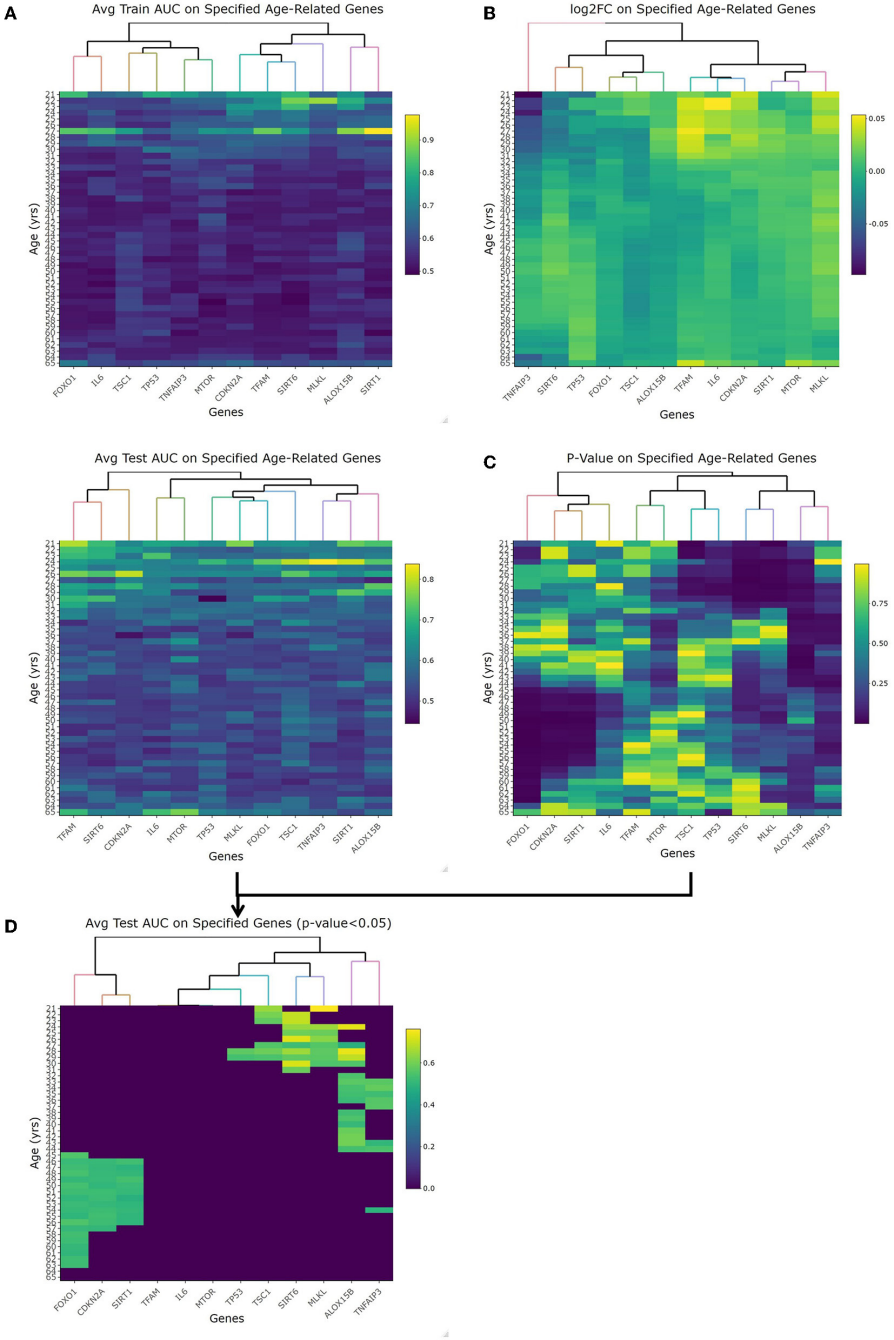
Empirical age threshold determination of 29 years old. **(A)** shows the average training and test area under the curve (AUC) results when using each genes (x-axis) to predict old/young with the cutoff equal to each year (y-axis). This orientation (genes/age on x-/y-axis) is used in each heatmap. **(B)** shows the log2 fold change of each gene at each age. With no magnitude greater than 2, we rely on AUC and *p*-value to determine age cutoff. **(C)** is the *p*-value of each gene at each age using the student *t*-test comparison. **(D)** is overlapping the test AUC and *p*-value so it is easier to see which genes are both significant and predictable of age. This is the heatmap used to choose 29-years-old as our cutoff given the most amount of significant genes that are also able to accurately predict young vs. old.

The first topic of debate to clarify was the threshold between what's considered an old or young patient. With these datasets including a large chronological range, linearized models fit to each year as a cutoff was evaluated by looking at the respective *p*-value, fold change, and accuracy with regard to AUC-ROC. The heatmaps for the *p*-values and log2FC from the *t*-test along with the train/test AUC-ROC heatmaps of the 12 previously mentioned genes are shown in Figure 2. Especially looking at the averaged AUC-ROC heatmaps (Figure 2A), it shows an increase in predictability at a younger age before dipping at middle-aged cutoffs and returning to a significant level with older values. Since no gene at any tested age cutoff showed a significant fold change with an absolute value greater than 2 (Figure 2B), the decision relied on the AUC-ROC of significantly different genes. The main portion to draw the reader's attention to is Figure 2D, this overlays the *p*-value with the average test AUC values to highlight the predictability of only the genes that were significantly changing at those age cutoffs. There were more genes of significant *p*-value (5 of 12 shown in Supplementary Figure 2B) as well as a higher average AUC (0.672) when “young” is classified as those with a chronological age equal to or less than 29 years and “old” classified as greater than young. This is our empirical definition of age cutoff or threshold that will be used to treat age as a categorical variable.

### 3.2. Statistical analysis and interaction modeling

**Figure 3:**
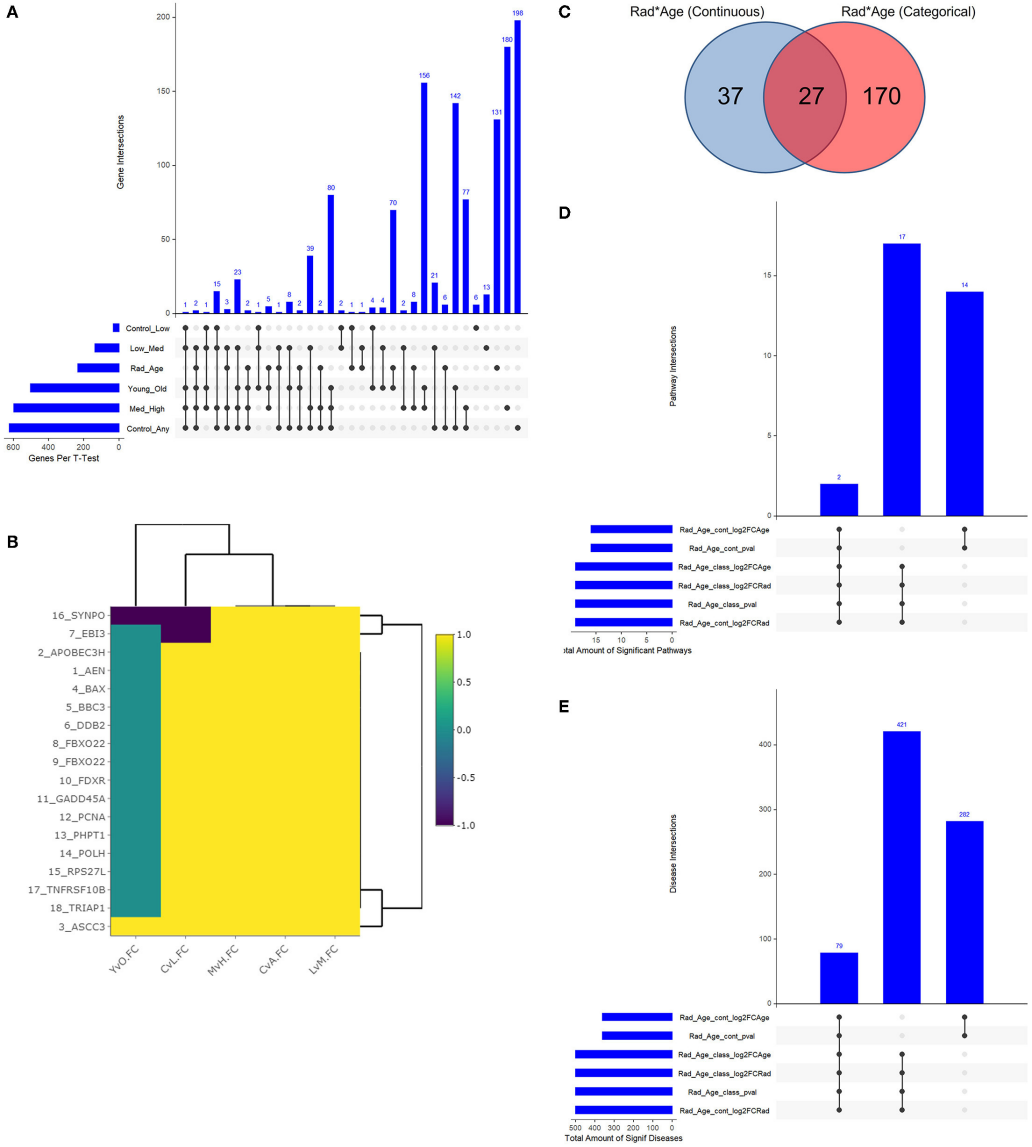
Resulting Venn diagrams and trend plot. Because of the large number of test cases, bar plots via UpSetR are used in place of traditional Venn Diagrams where applicable. **(A)** shows the multi-category overlap in significant genes between *t*-tests of increasing radiation exposure, age as a categorical variable, and the rad-age interaction test. While no gene was found in all test cases, 17 genes were common in many and explored more in the discussion as well as the next plot. **(B)** shows the up- and downregulation of the 17-overlapping *t*-test genes from **(A)** to emphasize fold change trend with increasing radiation exposure in relation to age. With yellow showing increasing gene expression, we mainly see an increase in expression with an increase in radiation exposure save a few genes which have a more detailed discussion in the manuscript. **(C)** shows the overlap between interaction models to identify 234 unique genes. This gene list is the basis for functional analysis in IPA to produce the remainder of these subfigures. **(D)** shows the overlap in significant pathways from the interaction test gene lists using IPA. PRPP biosynthesis and IL-1 signaling are the two common and discussed in detail in the text body. **(E)** shows the overlap in significant diseases from the same gene lists using IPA. A common theme of diseases deal with health issues in high proliferating cells and cancers of multiple tissue types.

With a young/old threshold of 29 years old, a student's *t*-test was conducted as described on respective datasets comparing means of two populations: control vs. any radiation, control vs. low radiation, low vs. medium radiation, medium vs. high radiation, and young vs. old. Interaction models were created using age as a categorical and continuous variable with radiation to explore dependent changes in gene expression. The significant genes with a false discovery rate (FDR) adjusted *p*-value of less than 0.05 of the seven described cases (combined 234 unique genes from the interaction models) were compared to see if there is any overlap in areas of interest in Figure 3A. Given numerous combinations of test conditions, comparisons are shown in an alternative representation to Venn Diagrams using the UpSetR v1.4.0 package within R. In Figure 3B, we viewed gene regulation direction for the 17 genes (18 probes) common amongst the *t*-test. From it, we see a trend of continued upregulation in gene expression as radiation levels increase in 15 of the 17 genes, the other two (*SYNPO* and *EBI3*) saw initial downregulation with low levels of radiation exposure before a continual upregulation with increased exposure levels. Of note with changes in expression for radiation and age are *ASCC3* and *SYNPO*. *ASCC3* is involved in nucleic acid unwinding and associated with homology directed repair and was observed to increase with age to corroborate this association (46). *SYNPO* is involved in actin-based cell shape and motility and was downregulated with age and from no- to low-radiation levels exposure (47). The other 15 genes show no directional change with age. Additionally, there are seven genes with various combinations of overlap between the *t*-test results and the interaction models that seem to support our association: *ANKRA2* is involved in enzyme binding activity and regulates histone deacetylases (48); *BRMS1L* is a breast cancer suppressor gene that is also a component of histone deacetylase complexes (49); *HADH* functions in mitochondria to catalyze oxidation (50); *POPDC2* is a membrane-associated protein in skeletal and cardiac muscle that is associated with regulating heart rate (51); *SAMD3, SLC4A11*, and *VAMP4* with lesser supporting literature.

### 3.3. Functional analysis

The two interaction models generated separate gene lists that were used for functional analysis following Figures 3C–E. When using age as a continuous variable, there were 64 significant genes, and 197 genes were significant with age as a categorical variable (Figure 3C). The respective *p*-value, log2FC(radiation), and log2FC(age) were all used to create a preranked list entering IPA. The results were 33 significantly expressed pathways (Figure 3D) associated with 782 diseases with list intersection (Figure 3E). We show dot plots for both pathways and diseases in Supplementary Figure 3. Of the 19 overlapping pathways, the two captured regardless of ranked list are IL-1 signaling and PRPP biosynthesis while other reoccurring pathways included dolichyl-diphosphooligosaccharide and creatine-phosphate biosynthesis. Of the 79 overlapping significant diseases, we see a common trend of cancers among high proliferating cells that can be expected from both older age and radiation exposure separately. While the top 20 diseases are shown to primarily be cancers of the digestive system, other diseases flagged as significant include non-melanoma tumors, extracranial tumors, head and neck tumors, pelvic cancer, breast cancer, and general carcinoma.

### 3.4. Clinical potential

**Figure 4:**
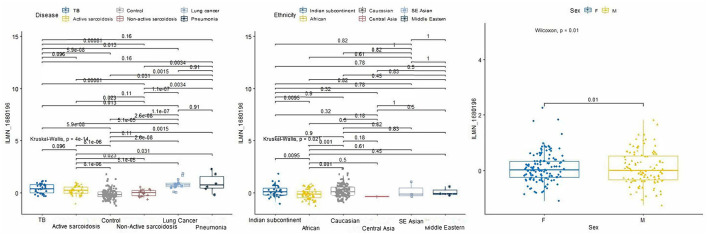
*P*-value boxplots of our most significant lung disease predictor gene: *LAPTM4B*. When using our rad-age gene list to attempt to differentiate lung disease of a separate dataset (*p* = 4e-14), this gene had a significant interaction (*p* = 0.004) when also accounting for sex (*p* = 0.01) and given ethnicities (*p* = 0.021). While this is not intended to have immediate effect on the community for biomarker identification, this does show that rad-age genes can supplement disease risk assessment in areas that may be independently associated with radiation exposure or aging while also accounting for external clinical factors.

From the 234 unique genes generated from the rad-age interaction tests, 10 genes were statistically significant when either looking at pairwise disease, ethnicity, or sex comparison across train, test, and validation subsets of Data-D1: *ASTE1, ATP2A3, CDYL2, LAPTM4B, PMS2P1, PXYLP1, ROM1, SLC37A3, SRPK1*, and *ZNF469*. Tables of these comparisons are given in the Supplementary material. When looking at a more detailed interaction between clinical factors, three showed significant relations: *LAPTM4B, ROM1*, and *SRPK1*. We want to highlight *LAPTM4B* (probe ID ILMN_1680196) which was the only gene to have a significant *p*-value (*p* = 0.004) when looking at the interaction among all three variables: disease, ethnicity, and sex. A box plot breakdown of this gene is shown in Figure 4 to highlight pairwise *p*-value and significance across each clinical factor. Meanwhile, *SRPK1* (probe ID of ILMN_1798804) was significant with disease and sex and *ROM1* (probe ID ILMN_1723743) was only significant with disease differentiation.

## 4. Discussion

Let us emphasize the importance that chronological age is used for this study under the assumption that biological age is relatively close to chronological age and attempting to estimate biological age is out of the scope of this article. Instead, finding a reasonable cutoff for chronological age gives a statistically significant foundation for future study starting points. The reasoning behind empirically defining a cutoff age is due to the lack of consensus on what a chronological cutoff age would be. For that purpose, these results support a continued use of a 29-year-old threshold delineating young from old for subsequent analysis. We admit this process is limited and an initial generalization of defining such a term. However, it provided a baseline to allow continuous and categorical analysis of a rad-age interaction. Ideally, a future study would run an identical approach on a much larger sample population while adjusting for clinical factors that were unavailable to our dataset such as sex and ethnicity. A multi-category cutoff of biological age depending on the patient is more reasonable given the changes in estimation with respect to such factors.

After defining a chronological age cutoff, the following becomes an effort to corroborate a rad-age association without necessarily identifying anything new or unknown to the community. This is done using a genomics only foundation to understanding functional relevance via pathway and disease analysis. Capturing 234 statistically significant genes are used for pathway and disease discussion, while the subset of 17 presented earlier starts painting the picture. Of the 15 genes that changed with radiation level but not age, seven were found amongst *t*-tests and four are mentioned to have supporting literature in their association to radiation exposure. Interestingly, *ANKRA1* and *BRMS1L* are involved in histone deacetylation while *HADH* functions in mitochondria to catalyze oxidation. While the gene expressions did not show change with age in our dataset, their functions are closely tied to biological aging processes. Both histone modification and oxidative stress are ongoing fields of study in aging with recent studies on using findings for understanding subpopulation aging and cancer therapy (52–54). It could be part of a future study with additional resources to see if these genes are a point of epigenetic change for biological age estimation.

As we aim to supplement a rad-age association, lets take some time to dig into the two genes that saw change in expression with respect to both age and radiation exposure: *SYNPO* and *ASCC3*. Although *SYNPO* is loosely involved, it's role in cell motility has an association with biological aging. As cells age, they may experience a decline in the ability to move, migrate, and respond to stimuli in their environment. This decline in cell motility can contribute to changes in tissue organization and function, as well as to the development of age-related diseases (55). Several factors are thought to contribute to the decline in cell motility with aging, including changes in the cytoskeleton and the cellular microenvironment. In particular, changes in the levels of matrix metalloproteinases (MMPs) and their inhibitors, as well as changes in the levels of extracellular matrix (ECM) components, can affect cell motility (56). In addition, oxidative stress, which is a hallmark of aging, can also lead to changes in cell motility. For example, oxidative stress can damage the cytoskeleton and the cellular machinery that is necessary for cell migration (57). With regard to the role of *ASCC3*, the relationship between nucleic acid unwinding and biological aging is not well-understood. However, there is some evidence to suggest that changes in the regulation of DNA unwinding and replication may contribute to aging and age-related diseases. Nucleic acids, such as DNA, are tightly wound and packaged in the nucleus of cells, and must be unwound to be transcribed and replicated. The process of DNA unwinding is tightly regulated and controlled by a variety of enzymes and factors, including helicases and topoisomerases (58). Aging can lead to changes in the regulation of DNA unwinding and replication, which can contribute to genomic instability, cellular senescence, and age-related diseases (59). For example, aging can result in changes in the levels of enzymes involved in DNA unwinding, leading to alterations in the rate and accuracy of DNA replication (60). Additionally, oxidative stress can cause DNA damage and lead to alterations in the activity of enzymes involved in DNA unwinding and replication (61). While the genes alone give us confidence in a basic genetics-based association, it also lends a hand to continued research on epigenetics to look at histone modification.

After running IPA, the respective dot plots showed high gene ratio and significant pathways of mention to be IL-1 signaling, PRPP biosynthesis, and RhoA signaling. Seeing IL-1 signaling is a catch-all pathway in our associative study since IL-1 is a pro-inflammatory cytokine that plays a central role in the regulation of the immune response and the maintenance of tissue homeostasis. IL-1 signaling pathway is activated by the binding of IL-1 to its receptor (IL-1R), leading to the activation of several intracellular signaling pathways, including the NF-κB, MAPK, and JAK-STAT pathways. In the context of aging, IL-1 signaling pathway has been implicated in the regulation of various aging processes, including cellular senescence, oxidative stress, and inflammation. Cellular senescence is a state of irreversible growth arrest that occurs in cells as a result of various stressors, including oxidative stress and DNA damage. IL-1 signaling pathway has been shown to play a role in the induction of cellular senescence by activating pro-inflammatory cytokine production and oxidative stress (62). We see that oxidative stress is mentioned again here with various age-related diseases, including cardiovascular disease, neurodegeneration, and cancer. IL-1 signaling pathway has been shown to contribute to oxidative stress by inducing the production of reactive oxygen species (ROS) and activating oxidative stress-responsive signaling pathways. Inflammation is another hallmark of aging and is associated with various age-related diseases, including cardiovascular disease, neurodegeneration, and cancer. IL-1 signaling pathway has been shown to play a central role in the regulation of inflammation by activating immune cells, inducing the production of pro-inflammatory cytokines, and triggering the release of various chemical mediators (63). While there was some variation, *GNA12* and *TOLLIP* were common in all gene lists that triggered a significant response via IPA in flagging this pathway being involved in regulating TOR and inflammation signaling.

Additionally, PRPP biosynthesis was also significant as it is an important intermediate in cellular metabolism. The PRPP biosynthesis pathway is an important metabolic process that involves the production of Phosphoribosyl pyrophosphate (PRPP), which is a key molecule involved in the synthesis of purines and pyrimidines, the building blocks of DNA and RNA. The pathway is regulated by multiple enzymes and factors, including PRPP synthase and inosine monophosphate dehydrogenase (IMPDH). The relationship between the PRPP biosynthesis pathway and aging processes is not well-understood, but some studies suggest that alterations in the pathway can contribute to aging-related cellular changes and diseases. For example, changes in the expression or activity of PRPP synthase or IMPDH can affect the balance between PRPP production and consumption, leading to imbalances in purine and pyrimidine metabolism and potentially contributing to age-related cellular dysfunction. However, more research is needed to fully understand the role of PRPP biosynthesis in aging processes, and to determine the specific mechanisms by which changes in the pathway might contribute to cellular aging and age-related diseases. *PRPS2* was in all gene lists and encodes a phosphoribosyl pyrophosphate synthase that plays a central role in the synthesis of purines and pyrimidines that catalyzes the PRPP reaction. Another pathway that overlapped many (but not all) cases was RhoA signaling which is key in cytokinesis, proliferation, and adhesion to MEF cells with multiple genes of this pathways being flagged.

As mentioned and expected, the majority of emphasized diseases included various cancers. The association between aging and radiation exposure with respect to cancer has been extensively studied. Radiation exposure is known to increase the risk of developing certain types of cancer, and this risk increases with age. The mechanisms underlying this association are not fully understood, but several potential mechanisms have been proposed, including oxidative stress, genomic instability, and inflammation. For example, one study showed that ionizing radiation can induce oxidative stress and DNA damage, leading to an increased risk of cancer (64). Another found that exposure to ionizing radiation can increase inflammation, which is known to play a role in the development and progression of cancer (65). Finally, another study explored the impact of radiation exposure on the genomic stability of cells by showing that exposure to ionizing radiation can cause DNA double-strand breaks, leading to chromosomal instability and an increased risk of cancer (66).

When looking to use the 234 genes to differentiate among lung diseases including cancer, 10 were statistically significant with one (*LAPTM4B*) being relevant across all ethnicity, sex, and diseases. *LAPTM4B*, also known as Lysosomal-associated protein transmembrane 4 beta, has been associated with various lung diseases. *LAPTM4B* is a lysosomal protein that plays a role in regulating the function of lysosomes, which are cellular organelles involved in the degradation and recycling of cellular waste. Studies have shown that alterations in the expression of *LAPTM4B* are associated with various lung diseases, including chronic obstructive pulmonary disease (COPD) and idiopathic pulmonary fibrosis (IPF). One such study found that *LAPTM4B* is upregulated in the lung tissues of patients with IPF, and that this upregulation is associated with increased oxidative stress and cellular injury (67), while another found that *LAPTM4B* is also upregulated in the lung tissues of patients with COPD and that this upregulation is associated with increased inflammation and oxidative stress (68). *LAPTM4B* has been shown to regulate the accumulation of oxidative stress and inflammation *in vitro*, as well as modulating the activity of enzymes involved in cellular senescence (69). However, more research is needed to fully understand the role of *LAPTM4B* in aging processes. Also of no great surprise is seeing *SRPK1* when evaluating lung disease relevance since this gene is associated with lung cancer and RNA binding and protein kinase activity. Future studies in this field would involve validating findings on a wider and larger population for potential use in a biosensor for monitoring lung disease development following radiation exposure.

## 5. Conclusion

The intent of this study was to use publicly available data from previously conducted, original studies to do secondary analysis with regard to associating aging processes with exposure to ionizing radiation. To avoid biological bias to underlying health conditions (cancer patients undergoing radiation therapy), we used datasets focused on blood samples from otherwise healthy patients who were exposed to radiation. Due to a lack of consensus or support from literature, a large number of control samples were used to define an age cutoff to create two populations between young and old which was empirically found to be 29 years old. With the combined datasets, student's *t*-tests were performed on a number of test conditions and more importantly a linearized model on the interaction test between age (both continuous and categorical) and radiation.

These statistical tests identified 15 overlapping genes with continued upregulation with increased radiation exposure, the interaction models identified 234 unique genes used for preranked analysis via *p*-value and fold change, and seven genes overlapped the interaction and statistical results emphasizing critical factors such as mitochondrial function, oxidation, histone acetylation, and cardiac function. From IPA via the preranked lists, two pathways were significant across our redundant preranked lists involving IL-1 signaling and PRPP biosynthesis; both of which corroborate a rad-age association. There's a multitude of overlaps in significant diseases with a fairly common trend being those associated with high proliferating cells as expected. Finally, evaluating these rad-age genes for indirect collaboration with other disease (namely those of the lung), 10 genes had significant independent pairwise comparison results, three of which with significant interactions and one with statistically significant differentiation, with disease, ethnicity, and sex taken into account, *LAPTM4B*.

The significant functional analysis along with the success rate using genes from the interaction model supplement theories on an association between ionizing radiation exposure and aging. These findings support the need for further association studies to confirm and serve as a foundation for biological age estimation and space studies. Should radiation have significant enough correlation, persons of the same chronological age will have a biological age variance explainable by measured radiation differences, and now a reference point to estimate BA without direct measurement.

## Data availability statement

The datasets presented in this study can be found in online repositories. The names of the repository/repositories and accession number(s) can be found in the article/Supplementary material.

## Ethics statement

Ethical review and approval was not required for the study on human participants in accordance with the local legislation and institutional requirements. Written informed consent for participation was not required for this study in accordance with the national legislation and the institutional requirements.

## Author contributions

NR performed the study conception and designing, collection of data, preprocessing of data, bioinformatic analysis and interpretation, and manuscript writing. SS performed bioinformatic analysis and contributed to the writing of the manuscript. KS contributed to collection of data and manuscript writing. JG contributed to preprocessing data. BB contributed to manuscript writing. DS provided guidance and financial support. SKS provided guidance to design study, collection of data, analyzed data, and contributed to the writing of the article. All authors contributed to the article and approved the submitted version.
