# Supplementary material for: Establishing a genomic radiation-age association for space exploration supplements lung disease differentiation
Source: Front Public Health. 2023 May 11;11:1161124. doi: 10.3389/fpubh.2023.1161124 (PMC10213902; doi:10.3389/fpubh.2023.1161124)
Supplement: Supplementary file 1 [file Data_Sheet_1.PDF]

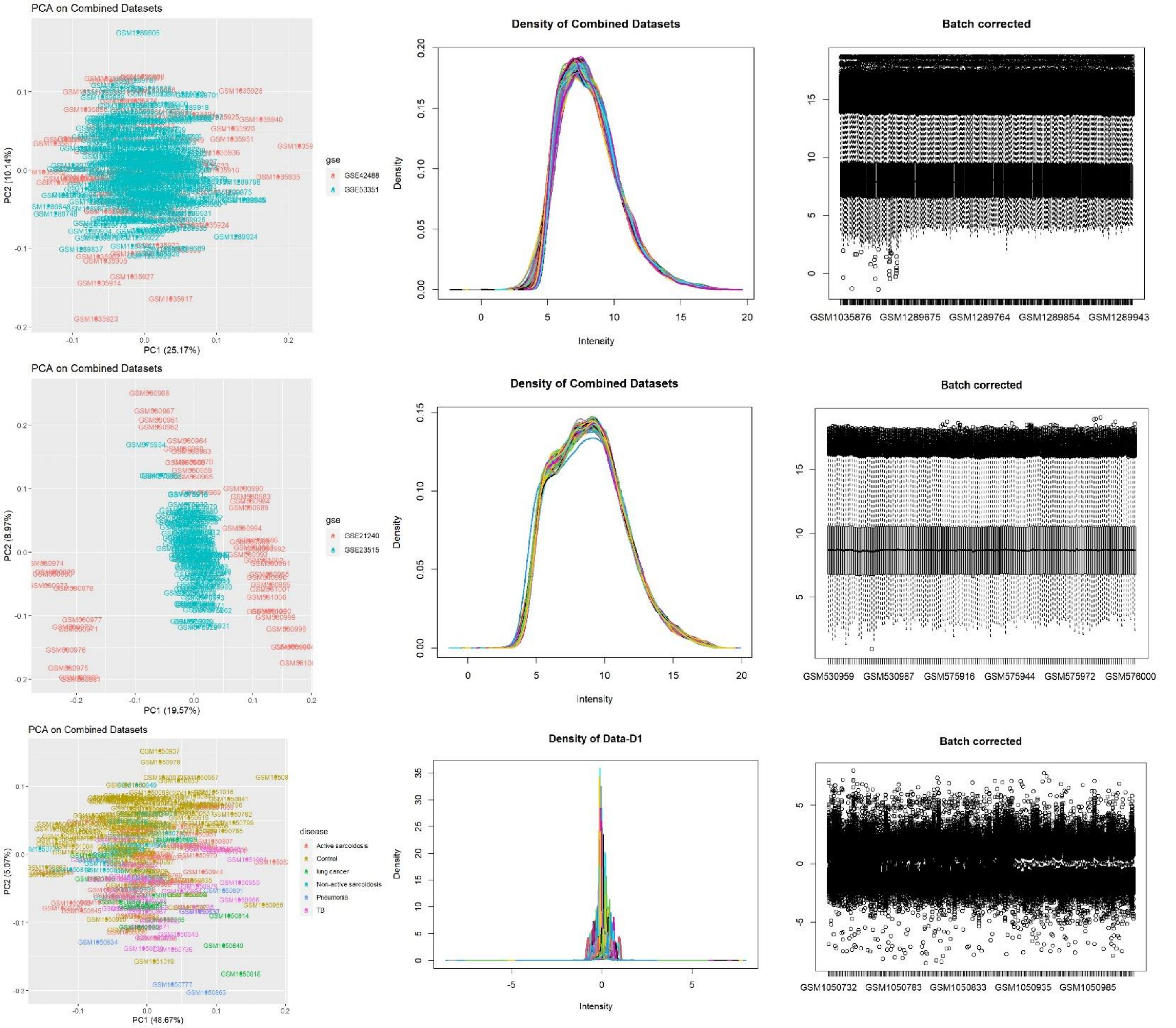

Figure S1 - Data Characterization. First (top) row shows the combined Data-A1 and Data-A2 plots with regards to PCA, gene expression, and boxplots of the combined set after removing the batch effect. Second (middle) row shows the same three plots for Data-B1 and Data-B2. Third (bottom) row shows Data-D1. T-test was run on combined data sets to compare the GSE identifiers to confirm no technical bias via high p-value between GSE sets.
