# Supplementary material for: Establishing a genomic radiation-age association for space exploration supplements lung disease differentiation
Source: Front Public Health. 2023 May 11;11:1161124. doi: 10.3389/fpubh.2023.1161124 (PMC10213902; doi:10.3389/fpubh.2023.1161124)
Supplement: Supplementary file 2 [file Data_Sheet_2.PDF]

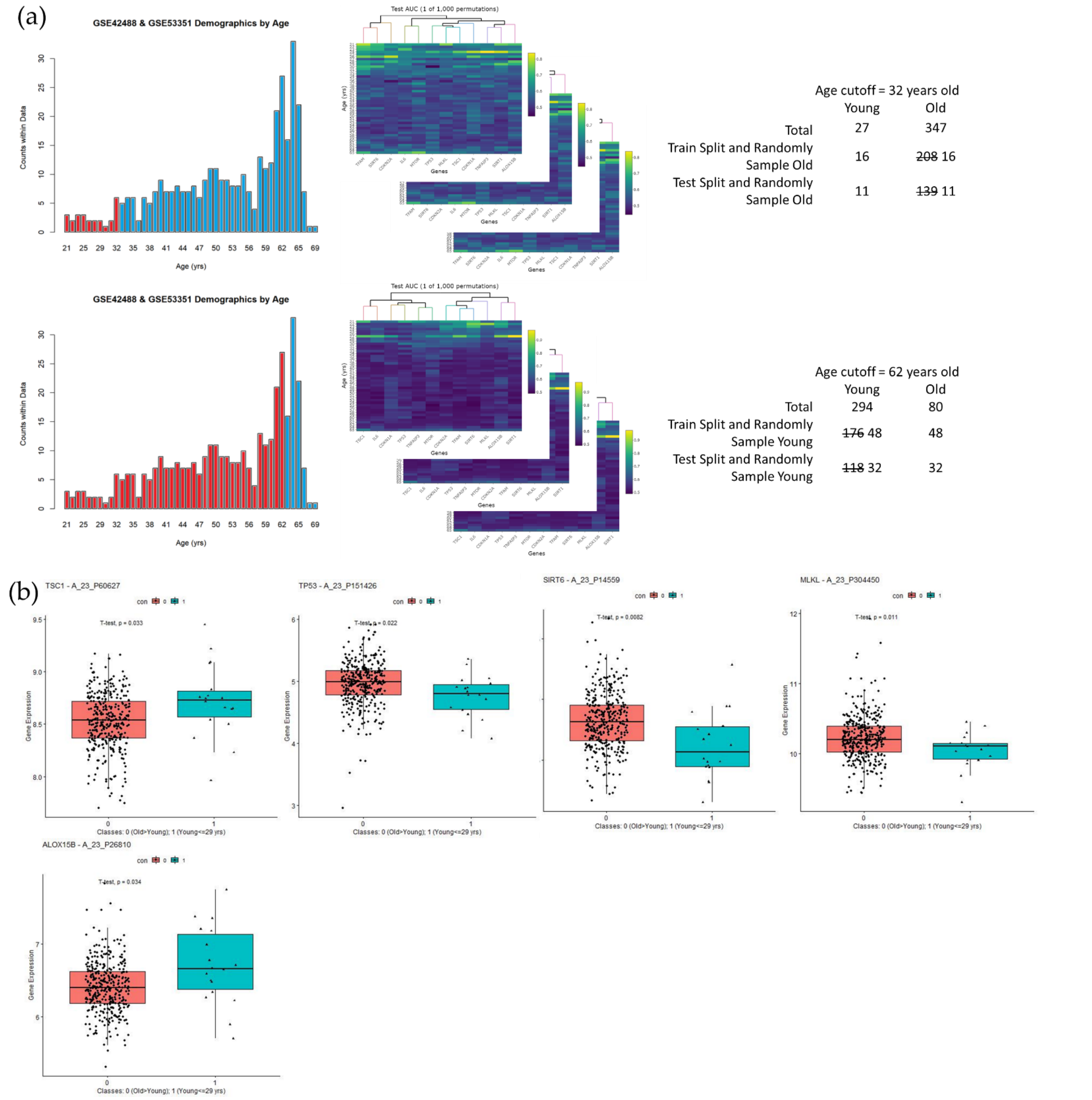

Figure S2 - (a) Shows the process followed when empirically defining a threshold between young and old patients. We randomly sample for a 50/50 split of the lesser category and ran 1,000 models on each gene to capture population characteristics. Results were the AUC-ROC of a linearized model working sequentially from 21 to 66 years old. As the evaluated age increases, "young" becomes the larger category in which we still randomly sample the lesser category (old) in order to maintain a 50/50 ratio. (b) Shows the respective boxplots 29-year-old cutoff from 5 of the 12 genes originally evaluated. These 5 are all statistically significant and had higher AUC when predicting age as a categorical variable for our reasoning to choose 29 as the threshold.
