# Supplementary material for: Establishing a genomic radiation-age association for space exploration supplements lung disease differentiation
Source: Front Public Health. 2023 May 11;11:1161124. doi: 10.3389/fpubh.2023.1161124 (PMC10213902; doi:10.3389/fpubh.2023.1161124)
Supplement: Supplementary file 3 [file Data_Sheet_3.PDF]

(a)

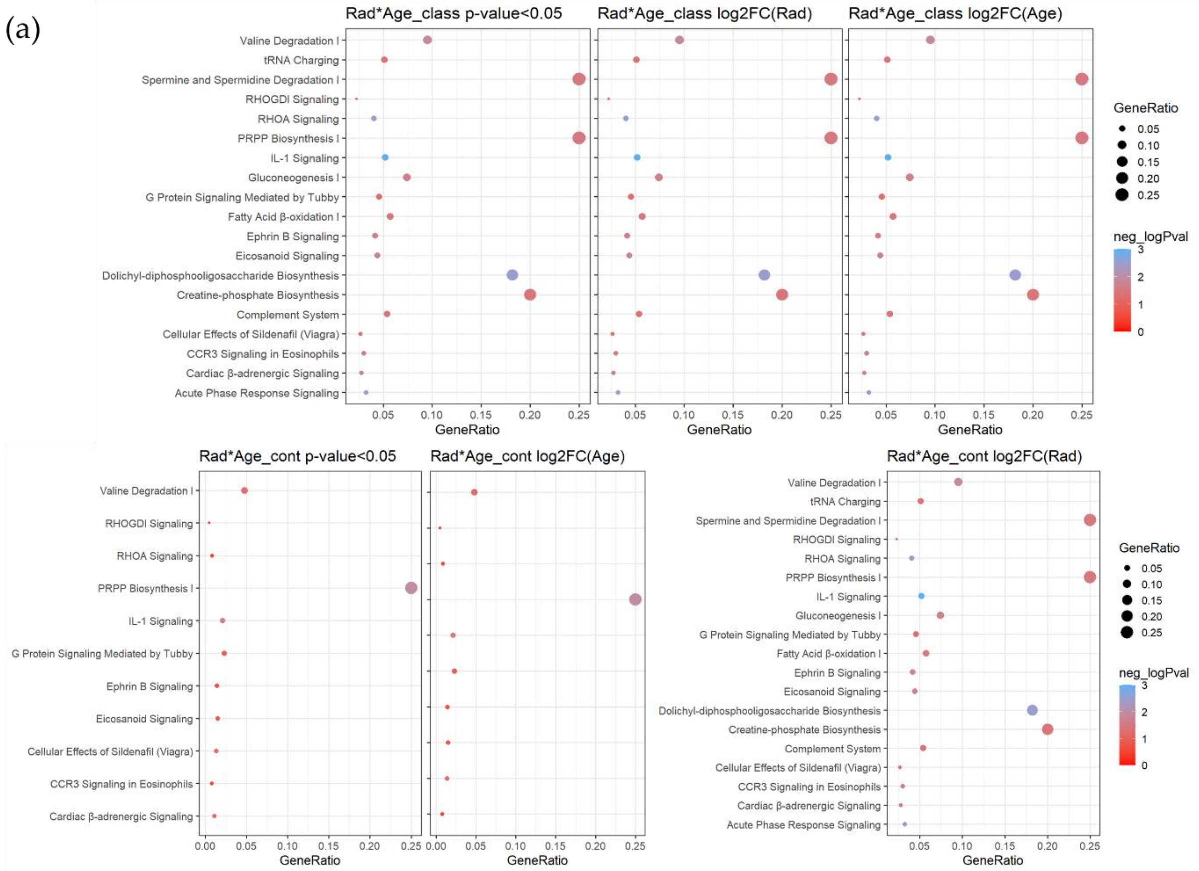

(b)

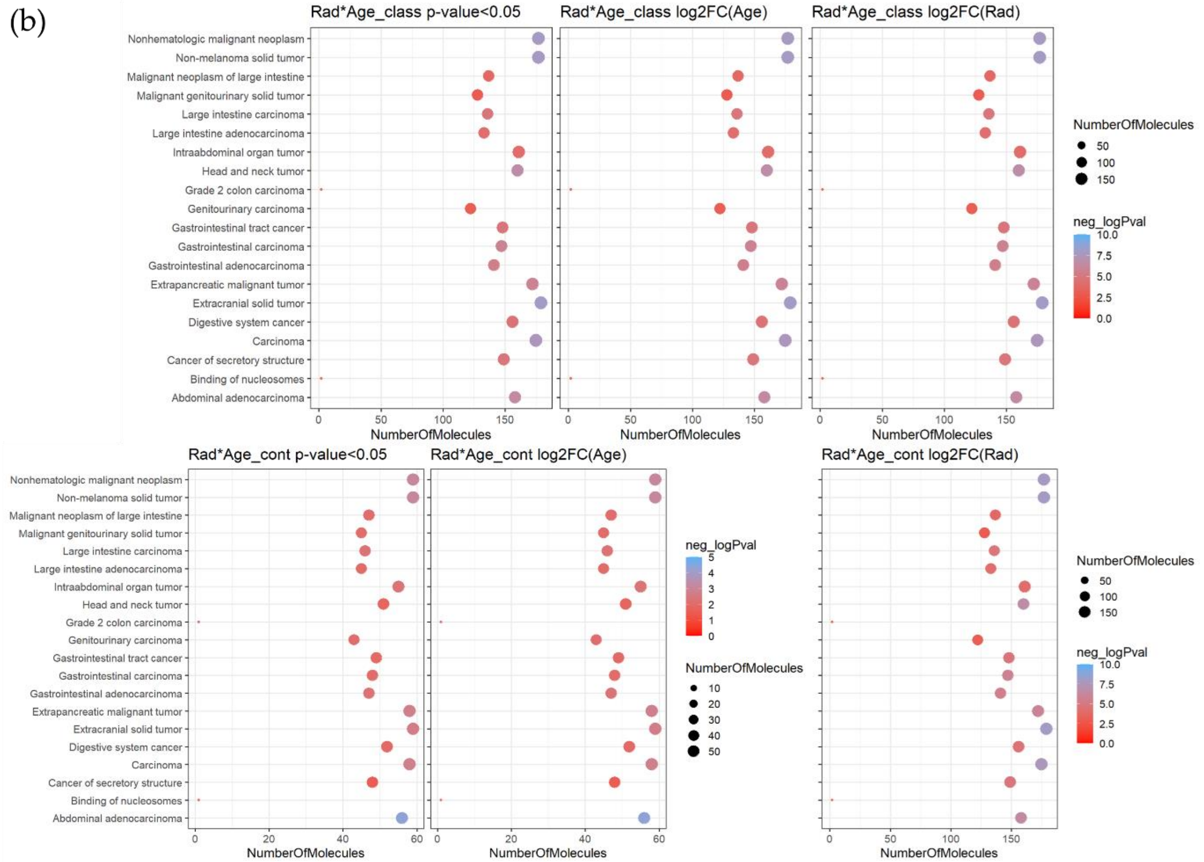

Figure S3 - Dot plots from IPA. (a) Shows the plotted significant of pathways from IPA from our rad-age interaction for both age as a categorical and continuous variable as well as across all redundant test cases to show corroboration. (b) Shows the plotted significance of diseases from IPA for the same test cases as (a). Redundancy across tests cases and age variable gives confidence in results seeing repeated pathways and diseases.
