# Supplementary material for: Establishing a genomic radiation-age association for space exploration supplements lung disease differentiation
Source: Front Public Health. 2023 May 11;11:1161124. doi: 10.3389/fpubh.2023.1161124 (PMC10213902; doi:10.3389/fpubh.2023.1161124)
Supplement: Supplementary file 4 [file Data_Sheet_4.PDF]

(a)

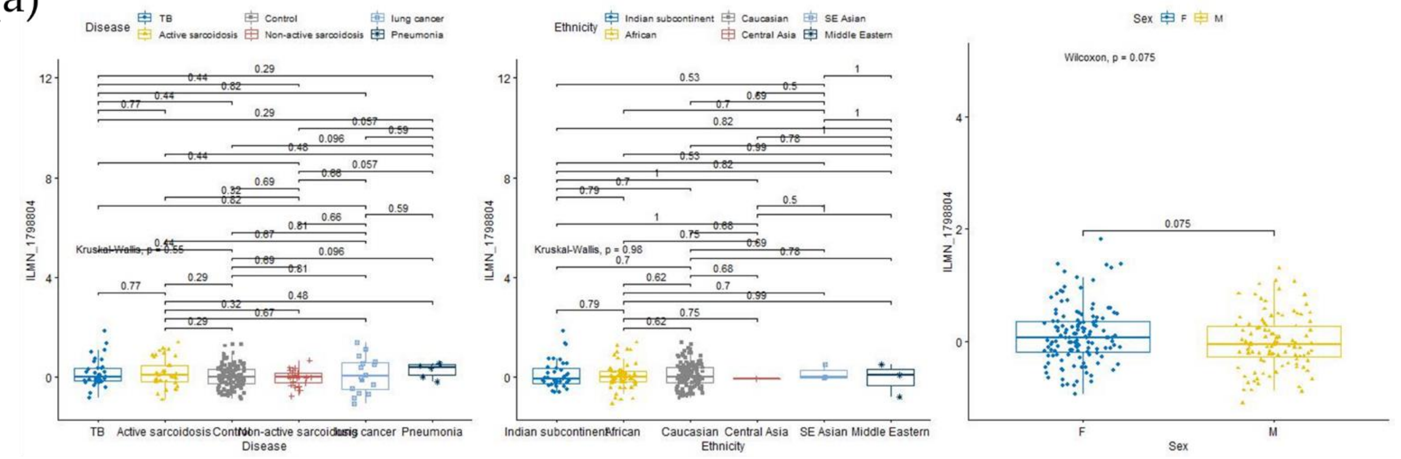

(b)

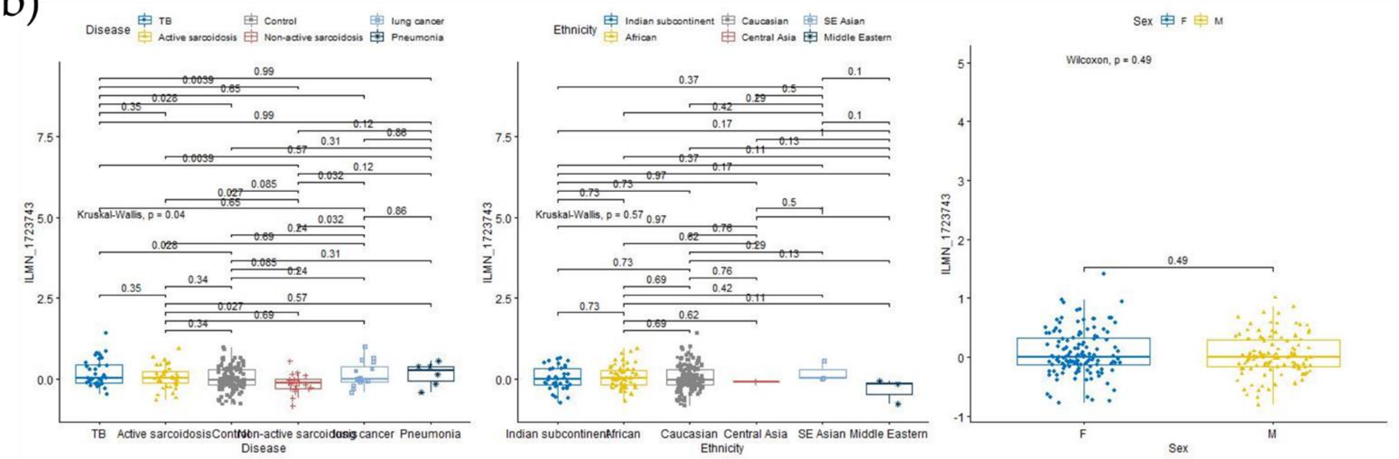

Figure S4 – Boxplots to show p-value significance across each disease, ethnicity, and sex. These two probes from our rad-age gene list originally showed potential in predicting disease while taking into account other clinical factors. At some point, these two did not pass tests. (a) Shows one such probe where predictability was good but without significant difference looking at ethnicity and sex. (b) Shows significance with disease but also not with ethnicity and sex.
